# Marked Erythroblast Haemophagocytosis in Association With Immunotherapy for Metastatic Melanoma

**DOI:** 10.1002/jha2.70069

**Published:** 2025-06-06

**Authors:** Tamasine Stewart, Joshua Casan, Surender Juneja

**Affiliations:** ^1^ Department of Pathology Peter MacCallum Cancer Centre Melbourne Victoria Australia; ^2^ Department of Diagnostic Haematology Royal Melbourne Hospital Melbourne Victoria Australia; ^3^ Sir Peter MacCallum Department of Oncology University of Melbourne Melbourne Victoria Australia

1

A 70‐year‐old male was being treated for metastatic melanoma at our institution and had received five cycles of nivolumab and relatlimab (a lymphocyte activation gene‐3 inhibitor). He developed acute agranulocytosis (neutrophils 0.0 × 10^9^/L) and anaemia (Hb 76 g/L) with inappropriately normal reticulocytes (absolute count 81 × 10^9^/L, 3.1%) and a normal platelet count (221 × 10^9^/L). He also developed a new erythematous rash, diarrhoea and left eye inflammation. Further blood tests demonstrated normal lactate dehydrogenase and haptoglobin, absence of spherocytosis or red cell agglutination and no evidence of classical haemophagocytic lymphohistiocytosis (HLH) (no fevers or splenomegaly, normal fibrinogen and triglycerides, ferritin 496 microg/L). A bone marrow aspirate and trephine biopsy demonstrated granulocytic maturation arrest at the promyelocyte stage (image D, Romanowsky stain, ×200). He also had significant phagocytosis of late erythroid precursors (up to 10 erythroblasts per histiocyte), (images A and B, Romanowsky stain, ×400) and platelets (image C, Romanowsky stain, ×400). Of note, there was no phagocytosis of granulocytes or their precursors. Overall, the multiple symptoms and granulocytic maturation arrest were in keeping with immunotherapy related adverse events.

His immunotherapy was withheld, and he received granulocyte colony stimulating factor which resolved his neutropenia.

HLH and agranulocytosis have been separately described previously in the setting of immunotherapy, but to our knowledge, this is the first case report describing both phenomena occurring concurrently in the one patient. Additionally, despite the striking erythrophagocytosis, there was unexpectedly no associated cytokine storm manifesting as clinical HLH, a syndrome that is becoming increasingly recognised in patients treated with immunotherapy. The mechanism for this, and the potential association between the immune‐mediated agranulocytosis, erythrophagocytosis and lack of clinical HLH, are unclear (Figure 1).

## Author Contributions

T.S, J.C. and S.J. conceived of the paper. T.S. and S.J. reviewed the investigations and captured the images. J.C. was responsible for the clinical care of the patient. T.S. wrote the manuscript in consultation with S.J. and J.C.

## Ethics Statement

No ethics approval is required at our institution for a case report.

## Conflicts of Interest

The authors declare no conflicts of interest.

2

**FIGURE 1:**
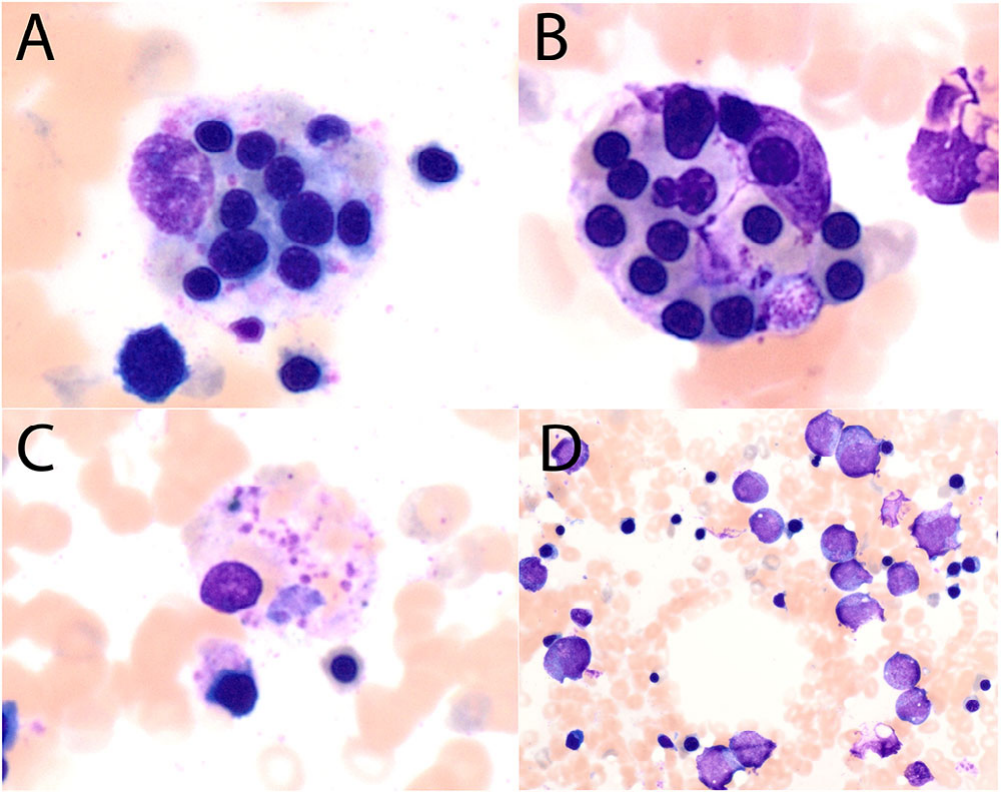
(A and B) Romanowsky stain, ×400. Phagocytosis of late erythroid precursors. (C) Romanowsky stain, ×400. Phagocytosis of platelets. (D) Romanowsky stain, ×200. Granulocytic maturation arrest at the promyelocyte stage.

## Data Availability

Data sharing not applicable to this article as no datasets were generated or analysed during the current study.

